# A Two-Level Ensemble Machine Learning Framework for OSA Classification Whilst Awake from Noisy Tracheal Breathing Sounds

**DOI:** 10.3390/s26041349

**Published:** 2026-02-20

**Authors:** Vahid Bastani Najafabadi, Walid Ashraf, Ahmed Elwali, Zahra Moussavi

**Affiliations:** 1 Department of Electrical and Computer Engineering, University of Manitoba, Winnipeg, MB R3T 2N2, Canada; bastaniv@myumanitoba.ca; 2 Department of Biomedical Engineering, University of Manitoba, Winnipeg, MB R3T 2N2, Canada; ashrafw1@myumanitoba.ca; 3 Department of Biomedical Engineering, Marian University, Indianapolis, IN 46222, USA; aelwali@marian.edu

**Keywords:** obstructive sleep apnea (OSA), wakefulness screening, ensemble machine learning, tracheal breathing sound, wavelet packet decomposition, bagged decision trees, probability-based voting, biomedical signal processing

## Abstract

**Highlights:**

**What are the main findings?**

- A two-level ensemble machine learning framework using awake tracheal breathing sounds achieved 77.1% accuracy, 84.3% sensitivity, and 59.9% specificity in a noisy recording environment.
- Stratifying nine sub-classifiers by anthropometric profiles and aggregating predictions using probability-based voting improved robustness under real-world noise.

**What are the implications of the main findings?**

- The results support feasible awake OSA screening in noisy, real-world recordings, motivating further validation on larger and more balanced cohorts.
- The framework’s high sensitivity suggests its effectiveness as a primary screening tool to prioritize at-risk patients for confirmatory clinical diagnosis.

**Abstract:**

Obstructive sleep apnea (OSA), defined by repetitive airway obstruction during sleep, is significantly underdiagnosed, mainly due to the resource-intensive and time-consuming nature of sleep assessment technologies. Machine learning analysis of the tracheal breathing sounds (TBS) whilst awake offers an alternative approach for OSA quick screening. This study aimed to address the challenge of wakefulness OSA detection using TBS recorded with an inexpensive microphone in a noisy environment. Data of 247 individuals with various degrees of OSA severity were analyzed. Recorded data were segmented into inspiration and expiration phases, followed by acoustic features extraction, feature reduction, and classification. A two-level ensemble architecture was implemented. Nine sub-classifiers were stratified by anthropometric profiles. Each sub-classifier was constructed as an ensemble of bagged decision trees, with a final prediction via probability-based voting. The proposed algorithm achieved an accuracy of 77.1%, sensitivity of 84.3%, and specificity of 59.9%. Although these results have lower performance than those obtained previously using a high-quality microphone in a quiet room, they demonstrate that acoustic OSA detection whilst awake remains feasible, even in very noisy environments. Nevertheless, microphone quality emerged as a key determinant of classification performance.

## 1. Introduction

Obstructive sleep apnea (OSA) is a sleep disorder characterized by repeated complete (apnea) or partial (hypopnea) cessation of the airflow during sleep [1], leading to disrupted breathing and fragmented sleep [2]. Excessive daytime sleepiness, fatigue, morning headaches, and mood changes are common clinical symptoms of OSA [3]. Severe OSA increases the risk of more serious disorders, including fatal and non-fatal cardiovascular events, compared to healthy people [4]. OSA is a very common disorder [5]; almost 1 billion people around the world are affected by sleep apnea, with the prevalence exceeding 50% in some countries [5]. Estimations show that 83.7 million people are experiencing OSA in the United States in 2024 [6]. Despite the OSA prevalence, it is significantly underdiagnosed and untreated [7].

Several risk factors contribute to developing OSA. Sex is a major risk factor; males generally experience more severe OSA compared to females [8,9,10]. Aging is another risk factor for OSA: older adults are at a higher risk of having OSA [11,12]. Increased body mass index (BMI) increases the risk of developing OSA [13,14,15]. Anatomical features related to the oropharyngeal airway, such as the Mallampati score (MpS), have also been shown to be significantly correlated with OSA severity [16], and is considered as an independent risk factor [17]. Daily habits such as smoking play a role in exacerbating sleep disorders. Current smokers are at greater risk of sleep disorders (i.e., OSA) than non-smokers [18,19,20]. In conclusion, OSA is a multi-faceted condition with many confounding variables contributing to its severity.

OSA detection relies on various clinical and diagnostic tools. The gold standard for detecting OSA involves polysomnography (PSG) [21], which is conducted in a sleep laboratory, where multiple physiological parameters, including brain activity, eye movement, oxygen saturation, etc., are monitored during overnight sleep [22]. This comprehensive data enables the assessment of OSA severity, which is measured by Apnea-Hypopnea index (AHI), defined as the number of apnea and hypopnea events per hour sleep. Despite the benefits of PSG, it is costly, time-consuming, and often not easily accessible to many patients [23,24]. Home sleep apnea testing (HSAT) technologies provide an alternative to in-laboratory PSG [25]. Unlike PSG, HSAT is performed in the patient’s home using portable monitoring devices that generally record a limited set of physiological signals [26]. HSAT, while more accessible, still requires overnight sleep data, and shows lower sensitivity than PSG for mild OSA [27]. These limitations have motivated the use of alternative approaches that do not require overnight monitoring. In routine clinical practice, initial screening often relies on validated questionnaires, such as the STOP-Bang questionnaire and the Epworth Sleepiness Scale (ESS). The STOP-Bang questionnaire estimates the risk of OSA based on BMI, age, snoring, and other subjective variables [28]. The ESS is also a subjective questionnaire that measures daytime sleepiness [29]. Although questionnaires are simple and useful for initial OSA risk assessment, their limitations in specificity, subjectivity, and reliance on symptoms make them inadequate as standalone diagnostic tools [25,30]. Given these drawbacks, researchers have explored additional markers that can be measured during wakefulness.

While various modalities have been explored, including speech analysis [31] and craniofacial photogrammetry [32], a recent publication has reviewed all the existing techniques to assess and screen OSA whilst awake [33]. One promising approach uses tracheal breathing sound (TBS) recorded during wakefulness to assess airflow resistance and estimate OSA severity, as tracheal sounds reflect the upper airway anatomy [34]. Formant analysis of TBS had revealed significant differences in specific frequency bands among mild and severe OSA patients [35]. Recent advancements have further demonstrated the potential of TBS analysis, with studies employing advanced machine learning models [36,37] and identifying specific acoustic biomarkers to achieve high diagnostic accuracy in controlled settings [38]. Building on this foundation, our group previously proposed an algorithm called AWakeOSA, which demonstrated the classification potential of TBS in OSA detection [39,40]. In that study, personalized subgroups based on anthropometric profiles (e.g., sex) were defined and separate classifiers were trained using TBS-derived features relevant to each sub-group [39]. The final decision was then determined by a weighted summation of the sub-classifier’s output. While this study proposed an accurate, reliable and rapid OSA screening tool, it relied on recordings collected in a quiet, highly controlled environment using an expensive professional microphone, conditions that are unlikely to be reproduced in routine hospital or clinical settings. Unlike anechoic chambers or soundproof booths, hospital rooms often have hard, flat surfaces, leading to reverberation and echoes. Conversations, foot traffic, ringing phones, and equipment sounds are several noises that occur in clinical spaces. The current study employs a lower-cost microphone and conducts data acquisition in a noisier environment, thereby simulating the realistic acoustic conditions encountered in hospitals and clinics. Specifically, the degradation in Signal-to-Noise Ratio (SNR) and the introduction of reverberation artifacts that may manipulate the nature of the TBS spectrum, present a substantial technical challenge for the established TBS feature extraction pipelines. This paper proposes a machine learning algorithm to address this environmentally challenging dataset by introducing a two-level ensemble architecture incorporating stability feature selection and bootstrap aggregating and investigates the extent to which diagnostic performance can be maintained under realistic acquisition protocols and inexpensive equipment.

## 2. Materials and Methods

### 2.1. Data Collection

Participants were recruited from individuals suspected of OSA who visited the Medical Medigas Center, Winnipeg, MB, Canada. Sleep apnea was assessed by either a portable PSG at their home or in a sleep lab at Misericordia Sleep Disorder Center (MSDC), Winnipeg, MB, Canada; all verified by respirologist. Individuals were excluded if they had any chronic respiratory disease, insomnia, or drug addiction. A total of 247 individuals aged 18–70 years old signed an informed consent form and were enrolled in the study. The study was approved by the Biomedical Research Ethics Board of the University of Manitoba.

TBS were recorded at the Remologie Sleep Clinic, Winnipeg, MB, Canada, during wakefulness in the supine position (with a pillow placed under the head) using a low-cost microphone (EM273) at a sampling frequency of 44.1 kHz. The microphone was inserted into a small chamber, placed over the suprasternal notch on the trachea. The participants were instructed to perform deep breathing, first through their nose for five breathing cycles, followed by five deep breathing cycles through their mouth, while wearing a nose clip. At the start of each maneuver, participants were instructed to hold their breath for a few seconds to record the background noise called silent period.

### 2.2. Data Preparation

Recordings with significant background noise (e.g., construction noise, speech) were excluded. The remaining recordings were segmented into inspiration and expiration phases by analyzing their logarithmic variance to detect the transitions between the phases. The SNR for each phase was quantified using the silent period of the corresponding signal. The raw dataset exhibited a mean SNR of 9.7 ± 5.6 dB for mouth breathings and 10.5 ± 5.7 dB for nose breathings, reflecting the challenging acoustic environment. Phases with SNR < 2 (approximately 3 dB) were excluded, particularly the inspiration and expiration phases adjacent to the silent period, to mitigate potential distortion of the acoustic characteristics. Subjects were excluded if their data contained fewer than two phases per maneuver. After exclusion, the final dataset yielded a mean SNR of 10.7 ± 4.2 dB for mouth breathings and 11.8 ± 4.8 dB for nose breathings. However, it is important to note that unlike laboratory settings where noise is typically low-level and stationary, our clinical recording contained high-amplitude non-stationary artifacts (e.g., ambient speech, hammer sounds). To ensure data quality, the mean power spectrum of the maneuvers was inspected for harmonic noise for each subject. After this refinement process, 228 subjects remained in the dataset. To facilitate binary classification and account for the potential overlap between the two groups (OSA and non-OSA) due to noise, a gap in the AHI threshold was implemented, thereby excluding 32 subjects and yielding a final sample of 196 participants, which each sample is defined as a unique subject with at least 2 phases per maneuver (e.g., mouth inspiration). Subjects with an AHI < 10 were labeled as non-OSA (*n* = 47), and those with AHI > 15 were labeled as OSA (*n* = 149). Table 1 summarizes the demographic and clinical information of the final cohort included in this study.

### 2.3. Data Preprocessing

**Figure 1:**
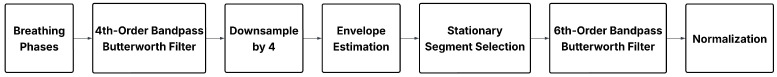
Preprocessing pipeline applied to breathing phases.

Breathing phases were passed through a 4th-order Butterworth band-pass filter (75–5000 Hz) as an anti-aliasing filter. The signals were then downsampled by a factor of 4 to reduce the computational cost. Stationary portions of the signals were extracted using the method described in [39]. The logarithmic variance was computed in 30 ms windows with 50% overlap to obtain the signal envelope. The stationary portion was defined as 50% of the signal duration centered on the maximum peak of the envelope. A 6th order Butterworth band-pass filter (75–3500 Hz) was subsequently applied to the stationary portion to mitigate any residual spectral leakage introduced by resampling. Finally, 8-sample moving average and standard deviation normalizations were applied to minimize intra-segmental and inter-segmental variations, respectively. The preprocessing pipeline is shown in Figure 1.

### 2.4. Feature Extraction

**Figure 2:**
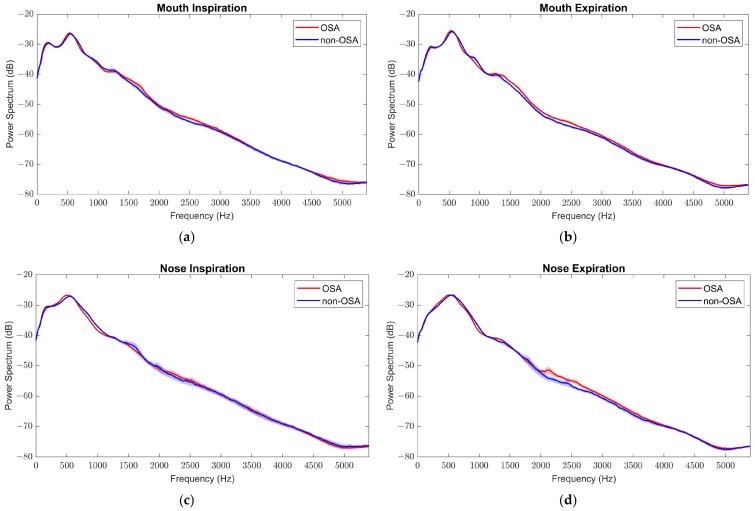
Mean power spectra (dB) of tracheal breathing sounds for OSA (red) and non-OSA (blue) subjects during (**a**) mouth inspiration, (**b**) mouth expiration, (**c**) nose inspiration, and (**d**) nose expiration maneuvers. Solid lines show the group mean, and shaded bands indicate ±1 standard error of the mean (SEM).

**Figure 3:**
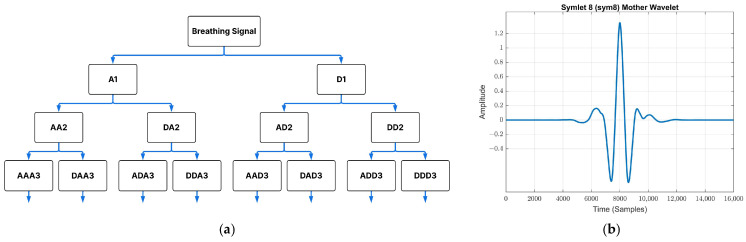
Wavelet packet decomposition tree (**a**) and Symlet 8 mother wavelet (**b**).

The mean power spectra of the two classes overlapped substantially across all frequency regions (Figure 2), indicating limited discriminatory power spectral analysis alone to capture the subtle acoustic features of OSA in a noisy clinical environment. The high degree of spectral overlap suggests that the discriminative information is not encoded in the average signal amplitude, but rather in localized non-linear irregularities and signal complexity. To overcome this limitation, Wavelet packet decomposition (WPD) was implemented as discriminative feature extraction technique, to move beyond 1D spectral plots and enable a multi-resolution analysis (Figure 3a). A Symlet 8 (sym8) mother wavelet function was selected to perform a 5-level decomposition, as it offers high symmetry, compact support, and a good balance between time and frequency resolution (Figure 3b). The corresponding WPD tree structure is shown in Figure 3.

Five-level WPD split the frequency range into 32 equally spaced sub-bands, each spanning a bandwidth of 172 Hz. Although both classes were highly overlapped in the frequency domain, the first 10 sub-bands below 2000 Hz were selected as the regions of interest for feature extraction. In total, 16 distinct features were extracted directly from the wavelet coefficients of 10 selected frequency sub-bands (0–1722 Hz) of each breathing cycle (e.g., a single mouth inspiration phase), as listed in Table 2. These specific features were driven by the underlying physics of respiratory airflow and airway anatomy. Statistical moments (e.g., Mean, Variance, Energy) were calculated to quantify the overall signal power, as the intensity of tracheal sounds is known to correlate directly with airflow rate and airway resistance [34]. Higher-order features (e.g., skewness, kurtosis) were included to quantify the non-Goussianity of the signal, as airway narrowing is known to induce turbulent airflow [34]. Spectral features (e.g., spectral centroid, spread) were extracted to capture variations in airway resonance [41]. Finally, information-theoretic features (e.g., Shannon, and Tsallis entropies) were utilized to characterize signal complexity, as it was determined that obstructive airway generates more disordered acoustic patterns compared to patent airways [42]. For each breathing maneuver (e.g., mouth inspiration), this resulted in 160 features (16 feature types × 10 sub-bands). These features were then averaged across all available breathing cycles within a specific maneuver. Finally, the feature vectors from the four maneuvers (mouth inspiration, mouth expiration, nose inspiration, and nose expiration) were concatenated to yield a vector of 640 features per subject (160 features × 4 maneuvers). All features were standardized using z-score normalization across all subjects.

### 2.5. Sub-Classifier Definition

**Figure 4:**
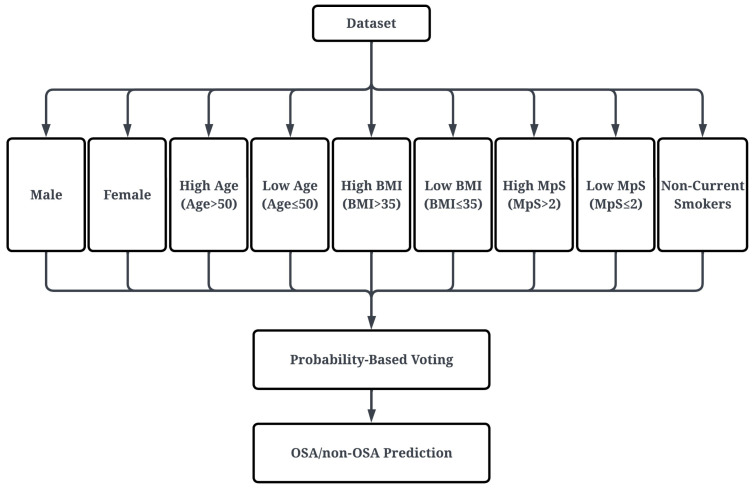
Ensemble voting across nine anthropometric sub-classifiers for final OSA vs. non-OSA prediction.

To address the population heterogeneity in OSA presentation, subjects were stratified into nine subgroups, as shown in Figure 4. These subgroups, based on components of the STOP-Bang questionnaire, included sex (male, female), age (age > 50, age ≤ 50), BMI (BMI > 35, BMI ≤ 35), MpS (MpS > 2, MpS ≤ 2), and smoking history (non-current smokers or former-smokers) [28]. The distribution of the subjects within each subgroup is presented in Table 3.

### 2.6. Training Strategy

A stratified 4-fold cross-validation was used to split the population into training and blind test sets for final model evaluation. For each subgroup, a dedicated sub-classifier was defined and trained exclusively on subjects from that subgroup within the training folds. Within each sub-classifier, an additional repetitive stratified *k*-fold cross-validation was applied to partition the training and validation sets; the validation sets were used to evaluate the feature selection and to tune hyperparameters. The value of *k* ranged from 3 to 6, depending on the number of non-OSA subjects available within the subgroup.

To address the high dimensionality of the feature space and ensure reproducibility, a hierarchical feature stability framework was implemented. While OSA and non-OSA subjects were highly imbalanced across most subgroups, feature selection was performed based on repeatedly balanced subsets. To establish intra-fold stability for each subgroup, we generated *B* = 1000 balanced subsets by combining all number of non-OSA subjects (minority class) with an equal number of randomly sampled OSA subjects (majority class). On each balanced subset, the minimum redundancy maximum relevance (mRMR) algorithm [43] was utilized to select the top 7 informative acoustic feature set *S*. The optimization objective of mRMR, which is to select features that maximize the mutual information with the target class *y* (relevance) while minimizing the mutual information shared with already selected features (redundancy), is defined as

(1) $$\underset{S\subset \;\Omega }{\max}\left[\frac{1}{\left|S\right|}\sum_{f_{i}\in S}I(f_{i};y)-\frac{1}{{\left|S\right|}^{2}}\sum_{f_{i},f_{j}\in S}I(f_{i};f_{j})\right]$$

where Ω denotes as the pool of 640 acoustic features, $\left|S\right|$ is the size of the selected subset, and $I\left(.;.\right)$ represents mutual information. For each iteration, only the top 7 features were retained. Features with an mRMR score below 0.01 were discarded, as low mRMR scores indicate limited informativeness. Following the 1000 iterations, a stability score was calculated for each feature based on its selection frequency across all subsets. The top 7 most stable acoustic features were then combined with three anthropometric features including neck circumference (continuous), snoring (Boolean), and receding mandible (3 classes), constructing the final 10 features to train the sub-classifier.

The repeated sub-sampling strategy employed for feature selection was replicated for model training to reduce bias toward the majority class and enhance robustness across subject combinations. Each sub-classifier consisted of an ensemble of multiple bagged decision trees, with each tree trained on a distinct balanced subset. All minority-class subjects were included in every subset, while an equivalent number of majority-class subjects were drawn without replacement from the remaining pool, ensuring that all majority-class subjects contributed to training. To cover all majority-class subjects, the minimum required number of models was determined by the ceiling of the class ratio: $N_{min}=\;\left\lceil {Ratio}_{OSA/non-OSA}\right\rceil$. To further maximize the exploration of subject combinations and enhance model generalizability, the final number of ensemble trees was set to $3\;\times \;N_{min}$.

Once the number of ensemble trees was determined and the features were selected, the training phase commenced. Each decision tree within the ensemble was trained on its own dedicated balanced subset using the selected feature vector. For each sub-classifier, predictions of the models on the validation sets were then aggregated across trees to evaluate the selected features and tune hyperparameters. A probability-based voting was implemented within each sub-classifier ($C_{k}$, where *k* denoted a specific anthropometric sub-classifier) to obtain a single prediction per subject. The average predicted probability of a subject x belonging to the OSA class, denoted as ${\hat{P}}_{k}\left(y=1\;|\;x\right)$, was computed across all bagged trees ($M_{k}$) as follows:

(2) $${\hat{P}}_{k}\left(y=1\;|\;x\right)=\frac{1}{M_{k}}\sum_{m=1}^{M_{k}}P_{m}(y=1\;|\;x)$$

To construct the final predictive sub-classifier utilizing the entire training set (both training and validation sets), a consensus ranking strategy was applied to the stable features identified across the inner cross-validation folds. For each cross-validation partition, a robust candidate set of 7 features was identified via the internal stability selection process described above. The candidate sets from all *k* inner folds (with their internal repetitions) were then pooled to form a global collection of high-stability markers. We calculated the recurrence frequency of each feature across these candidate sets. The top 7 features with the highest recurrence in this global pool were selected as the final acoustic features for that sub-classifier. These robust features were then combined with three anthropometric features to form the final 10-dimensional feature vector, as it has been determined that the inclusion of anthropometric features, in conjunction with acoustic features, yields a superior performance compared to acoustic features alone [39]. This feature combination also enabled us to compare the current study output with the previous proposed framework [40]. Subsequently, training and validation sets were concatenated to train the final sub-classifier using the same sub-sampling and ensemble strategy with this final feature vector.

**Figure 5:**
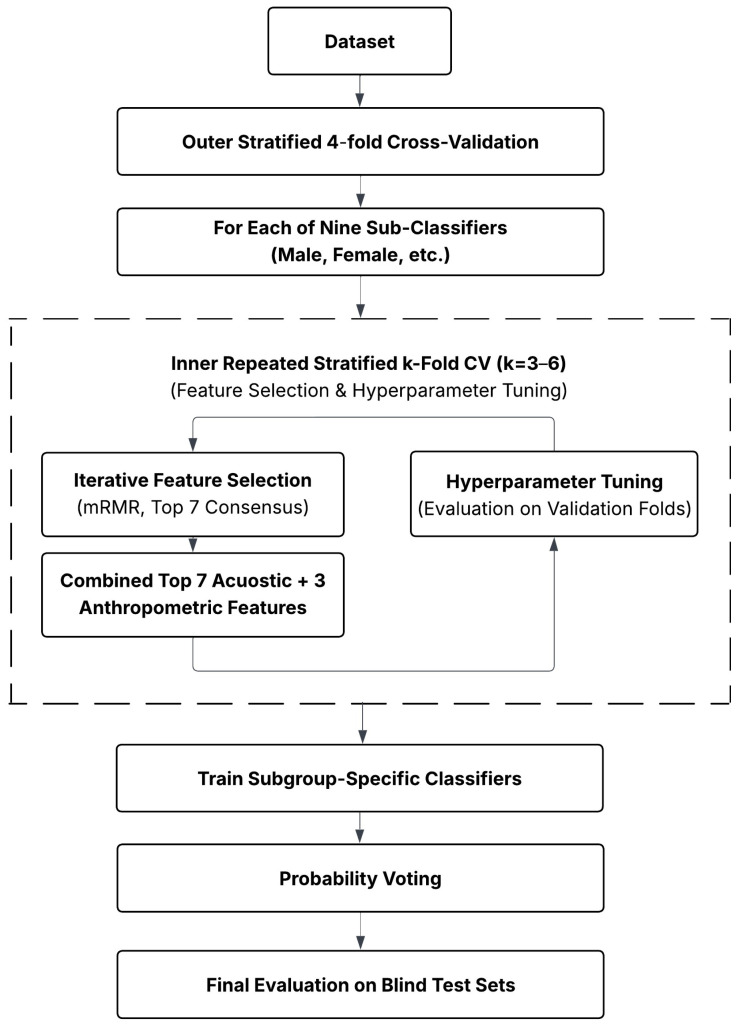
Schematic overview of the proposed Two-Level Ensemble Framework.

To obtain the final prediction, a dynamic ensemble selection was applied. The probabilities from all sub-classifiers relevant to the subject’s anthropometric profile (e.g., male, high age, high BMI) were averaged to produce the final probability ${\hat{P}}_{final}(x)$:

(3) $${\hat{P}}_{final}(y=1\;|\;x)=\frac{1}{\left|K_{x}\right|}\sum_{k\in K_{x}}{\hat{P}}_{k}(y=1\;|\;x)$$

where $K_{x}$ is the set of indices for sub-classifiers relevant to subject x. Subjects with ${\hat{P}}_{final}(x)$ < 0.5 were labeled as non-OSA, whereas subjects with ${\hat{P}}_{final}(x)$ ≥ 0.5 were labeled as OSA. Figure 5 provides a schematic overview of our entire algorithm.

Accuracy, sensitivity, specificity, and area under the curve (AUC) for each fold were computed to comprehensively assess the algorithm performance. The mean and standard deviation across folds were also reported to summarize variability. The balanced accuracy, defined as the average of sensitivity and specificity, was additionally presented to account for the class imbalance. An aggregated confusion matrix, obtained by pooling predictions across all folds, was used to detail the counts of false positives and false negatives, which offered insight into error patterns. All preprocessing routines, feature extraction procedures, and classification algorithms were developed and executed in MATLAB R2023b (The MathWorks, Inc., Natick, MA, USA, 2023).

## 3. Results

### 3.1. Final Predictive Feature Set

From the initial 640 acoustic features extracted from four breathing maneuvers (nose and mouth inspiration/expiration), the seven most frequently selected acoustic and three anthropometric parameters (neck circumference, snoring, and receding mandible) were used to train the sub-classifiers. Table 4 summarizes the final stable acoustic features selected across all sub-classifiers, defined as those selected in at least three out of four folds.

To evaluate the specific contribution of acoustic information relative to the three anthropometric input features, we analyzed the stability of the acoustic features within each subgroup. Rather than exhibiting random variation, which would be expected if the classification were driven primarily by the anthropometric inputs, distinct and high-stability acoustic features were observed across different subgroups. This systematic variation implies that the selected acoustic features provide necessary discriminative resolution that compliments, rather than duplicates, the baseline risk information captured by the anthropometric inputs. For example, in the male sub-classifier, f9 (spectral centroid in 0–172 Hz sub-band during mouth inspiration) was selected in all folds. The female sub-classifier frequently included f410 (spectral kurtosis) and f412 (spectral entropy) from the 861–1034 Hz sub-band during nose inspiration. This divergence suggests that while the anthropometric features (e.g., neck circumference) provide a baseline risk profile, the model explicitly requires distinct spectral patterns to accurately classify the subject. Similarly, the high age and low age sub-classifiers, features such as f616 (the median of nose expiration in the 1378–1550 Hz sub-band) and f522 (spectral kurtosis of the 344–516 Hz sub-band during nose expiration) were repeatedly selected. The high BMI sub-classifier consistently included f362, which quantifies the spectral kurtosis of the 344–516 Hz sub-band during nose inspiration, whereas the low BMI sub-classifier tended to favor mid to higher-frequency sub-band descriptors, particularly mean and spectra related features above 800 Hz. Both high MpS and low MpS sub-classifiers exhibited a preference for features from mid- to high-frequency ranges (around 1200–1700 Hz), underscoring the relevance of higher frequency content in these groups. For the non-current smokers sub-classifier, f599 (zero-crossing rate of the 1206–1378 Hz sub-band during nose expiration) and higher-order statistical and/or spectral features were frequently included, highlighting the role of temporal and spectral complexity in this subgroup. These findings indicate that the three anthropometric input features alone are insufficient for robust classification. The consistent selection of specific sub-band/descriptor pair serves as a complementary, high-resolution signal that distinguishes OSA from non-OSA individuals within the distinct sub-classifiers.

### 3.2. Performance Across Four Folds

The proposed classification algorithm was evaluated using repeated 4-fold cross-validation to ensure a balanced representation of OSA and non-OSA subjects in each fold and to assess its generalizability across different data splits. Table 5 presents the classification metrics across each fold, including balanced accuracy, accuracy, sensitivity, specificity, and area under the curve (AUC).

The algorithm achieved an average balanced accuracy of 72.1 ± 2.0%, slightly lower than the overall accuracy of 77.1 ± 3.1%. This difference indicates better performance on OSA subjects than on non-OSA subjects. The sensitivity of 84.3 ± 5.8% shows that the proposed algorithm correctly identified most OSA subjects, while the specificity of 59.9 ± 9.4% implies that the framework was less effective in distinguishing non-OSA subjects.

The mean AUC was 0.77 ± 0.01, indicating fair-to-good discriminative capability irrespective of the decision threshold. Sensitivity was consistently high across folds, while specificity exhibited greater variability, ranging from 72.7% in fold 1 to 50.0% in fold 4. This variability suggests that the non-OSA classification performance is more sensitive to the particular combination of training and testing datasets.

### 3.3. Probability-Based Voting vs. Majority Voting

Majority voting performance metrics are presented in Table 6. Majority voting attained a mean balanced accuracy of 71.2 ± 5.5% and an overall accuracy of 78.1 ± 3.1% across the four folds. The approach exhibited high and stable sensitivity (84.6 ± 1.3%); however, its specificity was comparatively low (57.8 ± 10.7). When comparing the two voting approaches, both achieved comparatively similar sensitivity; however, probability-based voting yielded greater specificity (by approximately 2.1%) and slightly improved overall discriminative capability. This indicates that probability aggregation better utilizes the confidence levels of the individual sub-classifiers, whereas majority voting weights all predictions equally. Therefore, probability-based voting may be preferable for screening, as it maintains good sensitivity while reducing the false alarm rate.

**Figure 6:**
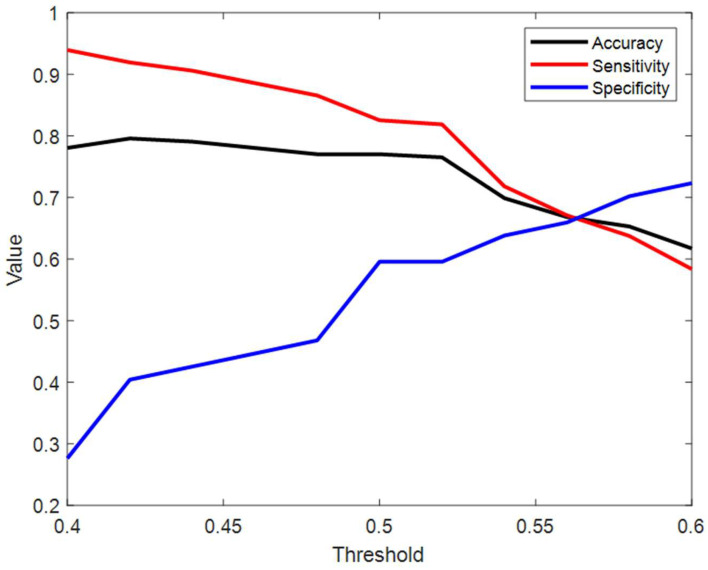
Performance trade-off analysis across varying probability thresholds, measured by accuracy (black line), sensitivity (red line), and specificity (blue line).

The decision threshold was also optimized to address the class imbalance and minimizing false negatives. Figure 6 illustrates the trade-off between accuracy, sensitivity, and specificity across thresholds ranging from 0.4 to 0.6. As shown, the default threshold of 0.5 yielded a high sensitivity (~84%) but moderate specificity (~60%). Increasing the threshold toward 0.55 improves specificity to approximately 65% but causes a sharp decrease in sensitivity to 68%. The crossover point, where sensitivity and specificity are balanced, occurs at approximately 0.56; however, sensitivity drops below 70% at this point, which is not optimal for a screening application. Consequently, the threshold 0f 0.5 was retained to prioritize the detection of true OSA subjects.

### 3.4. Leave-One-Subgroup-Out Analysis

To gain deeper insight into the relative contribution of each anthropometric subgroup to the final classification output, a leave-one-subgroup-out (LOSO) fusion analysis was performed. In this analysis, predictions of one subgroup (e.g., male, female) were removed at a time, and the final output was computed based on the remaining eight sub-classifiers as reported in Table 7. This approach facilitates the investigation of each subgroup’s effect by considering the variation in performance resulting from excluding its predictions.

Overall, removal of individual sub-classifiers substantially reduced the performance, indicating that each sub-classifier added unique and informative value to the overall ensemble. Removing the female sub-classifier, in particular, resulted in the lowest balanced accuracy rates (67.4%) and specificity (55.7%), suggesting that its predictive power is highly beneficial to the ensemble approach. Similarly, removing the high BMI sub-classifier lowered the balanced accuracy to 68.2%, consistent with the established association between obesity and OSA. Excluding either low age or high MpS sub-classifiers had smaller impacts with balanced accuracy remaining close to 71%, which may reflect possible redundancy or overlapping with other subgroups.

Interestingly, removing the non-current smokers sub-classifier resulted in a slight increase in sensitivity (85.2%), but a noticeable reduction in specificity (51.3%), which indicates its role in distinguishing non-OSA subjects and controlling false positive rates. Taken together, the LOSO analysis shows that while all sub-classifiers contribute to the ensemble’s robustness, classifiers linked with established OSA risk factors (sex, age, and smoking status) play a critical role in maintaining balanced performance.

### 3.5. Comparison with Previous Algorithms

In order to evaluate the proposed ensemble method, we compared its performance against three benchmarks: the STOP-Bang questionnaire, our previous AWakeOSA algorithm [40], and a global non-stratified baseline (a standard Bagged Trees ensemble without anthropometric stratification) using the same noisy dataset acquired in this study. Table 8 summarizes the results, showing that the proposed algorithm demonstrated a consistent superiority over all diagnostic methods.

As observed, although STOP-Bang achieved high sensitivity (93.6 ± 2.5%), it suffered from extremely low specificity (28.3 ± 3.1%), resulting in a large number of false positives. In contrast, the AWakeOSA algorithm produced higher specificity (59.1 ± 5.7%) but noticeably low sensitivity (37.8 ± 3.6%), leading to an unreliable OSA screening tool for our dataset. Crucially, the global baseline highlighted the necessity of the proposed two-level architecture. While the global baseline achieved high accuracy of 78.6%, this metric was misleadingly driven by a severe bias toward the majority class (OSA), failing to generalize to non-OSA subjects, resulting in a specificity of only 14.6%. The proposed framework achieved a balanced accuracy of 72.1 ± 2.0%, markedly higher than all comparators. By stratifying the problem into anthropometrically homogeneous subgroups, our framework successfully mitigated the class imbalance that caused the global baseline to fail, maintaining a robust trade-off between sensitivity (84.3 ± 5.8%) and specificity (59.9 ± 9.4%). This balance indicates that the proposed framework enables reliable OSA detection within an affordable and noise-tolerant experimental setup, outperforming questionnaire-based, previous acoustic-based, and global baseline methods.

### 3.6. Confusion Matrix Analysis

Further insight into the accuracy of the classification of the proposed ensemble algorithm was obtained by examining the confusion metrices of the test folds. The overall out-of-fold confusion matrix shows that the ensemble correctly identified 123 OSA cases and 28 non-OSA cases, while misclassifying 26 OSA and 19 non-OSA cases. These results translate into an accuracy percentage of 77.0%, a sensitivity percentage of 82.6%, and a specificity percentage of 59.6%, resulting in a balanced accuracy of approximately 71.1%.

To better characterize misclassified subjects, participants were divided into four groups based on their classification results: correctly classified as OSA, correctly classified as non-OSA, false negatives, and false positives (Table 9). Subjects correctly classified as OSA (*n* = 123) had significantly higher mean AHI (44.7 ± 28.0 events/hour) and higher BMI (36.8 ± 7.1 kg/m^2^) compared with correctly classified as non-OSA (*n* = 28), who had low AHI values (5.3 ± 2.6 events/hour) and a smaller neck circumference (38.3 ± 3.8 cm).

The false negative group (*n* = 26) had severe AHI values (32.3 ± 15.5), lower BMI (31.9 ± 5.2), and a relatively smaller neck size (38.5 ± 2.8 cm) compared with true OSA cases. False positives (*n* = 19) had mild AHI (6.3 ± 2.5), but their neck circumferences (40.3 ± 3.2 cm) and BMI (32.3 ± 7.1) were closer to the range of OSA, reflecting an anthropometric similarity with true OSA patients. In terms of sex distribution, the majority of false negatives were females (76.9%), while false positives were predominantly males (63.1%). Snoring remained high across all groups (>84%). The distribution of MpS and receding mandible also indicated overlap between misclassified and correctly classified groups. Overall, misclassification occurred primarily in subjects whose anthropometric features mimicked the opposing class, leading to acoustic patterns that confounded the framework’s decision boundary.

## 4. Discussion

This study introduces and validates an ensemble classification framework for OSA detection during wakefulness using a low-cost microphone in a noisy environment, utilizing tracheal breathing sounds signals from both mouth and nose breathing maneuvers, combined with anthropometric information. The results confirm the framework reliability in Awake OSA screening in acoustically challenging conditions. Several important arise from these results and are discussed below.

### 4.1. Feature Relevance and Physiological Interpretability

The feature selection analysis reveals that, although specific feature sets varied across folds and anthropometric subgroups, certain frequency-band features recurred frequently, indicating a reproducible discriminative pattern. This result suggests that while the implemented methodology successfully extracted discriminative patterns representative of physiological information, features are highly linked to the combination of subjects in the training set due to various selected feature sets. Notably, the feature selection analysis revealed a balanced distribution of features between inspiratory and expiratory phases, suggesting that both parts of the respiratory cycle contribute effectively to discriminative performance. The selection of inspiratory features (e.g., f9) aligns with the established pathophysiology of OSA, where airway collapse and wall vibration are driven by negative intraluminal pressure during inhalation [44,45]. Simultaneously, the equal recurrence of expiratory features indicates that valuable acoustic information is further present during exhalation due to greater turbulence, and flow–structure interaction during airway reopening [46]. In addition, the selected expiratory features are aligned with a study indicating that expiratory flow limitation (EFL), characterized by upper airway narrowing during expiration, is highly prevalent in OSA patients [47].

At the level of frequency composition, the selected features included both the lowest frequency band (0–172 Hz) and mid-to high-frequency ranges (344–1548 Hz). We found that low-frequency measurements (e.g., f9) captured the fundamental resonance of the upper airway, which functionally linked to anatomical dimensions [48]. Conversely, mid- and high-frequency features align with the physics of turbulent airflow; as the airway narrows, localized flow velocity increases, shifting acoustic energy toward higher frequencies [34]. Therefore, the model appears to consider a combination of low-frequency structural resonance and high-frequency turbulent flow markers to distinguish OSA subjects from non-OSA subjects.

The analyses performed on a subgroup level confirmed the occurrence of physiologically important patterns in the sets of features. The most commonly selected feature among male participants was f9 (mouth-inspiration, 0–172 Hz, spectral centroid), representing overall inspiratory load and the propensity for airway collapse even in the presence of increased airway caliber [49]. By contrast, female subgroups emphasized f410 (nose-inspiration 861–1033 Hz, spectral kurtosis) and f412 (nose-inspiration 861–1033 Hz, spectral entropy) as best features, corresponding to decreased upper-airway dimensions and an enhancement of higher-frequency irregularities related to inspiration [50]. For high-BMI subgroups, selected feature with the highest consistency was f362 (nose-inspiration 344–516 Hz, spectral kurtosis), representing the effects of mid-frequency nasal-inspiratory irregularity most likely due to obesity-induced soft-tissue loading [51]. By contrast, low-BMI subgroups highlighted primarily f410 (nose-inspiration, 861–1033 Hz, spectral kurtosis) together with f113 (mouth-inspiration, 1206–1378 Hz, mean amplitude), suggesting the relevance of nasal-inspiration irregularity at higher frequencies, together with the temporally specific nature of oral inspiration in leaner phenotypes. Likewise, age-related sub-classifiers agreed in consistently emphasizing nasal-expiration features f522 (nose-expiration, 344–516 Hz, spectral kurtosis) and f616 (nose-expiration, 1378–1550 Hz, median amplitude), posing the possibility that expiratory turbulence could represent an indicator of age-related changes in tissue compliance [52]. Finally, the non-current/former smoker sub-classifier included mostly f599 (nose-expiration, 1206–1378 Hz, zero-crossing rate), suggesting that temporally irregular patterns of airflow are particularly relevant when smoking-related airway inflammation is absent.

Crucially, this subgroup-specific heterogeneity provides strong evidence that the classification is not driven primarily by the three anthropometric features (neck circumference, snoring, and receding mandible). If the model relied dominantly on these anthropometric features, the selected acoustic features would likely exhibit random variation or low stability, acting merely as noise. Instead, the emergence of distinct high-stability acoustic features tailored to specific anthropometric profiles confirms that the acoustic data provides necessary discriminative ability that the anthropometric features cannot capture alone. The anthropometric subgroups function as context-setting priors, while the specific acoustic features provide the orthogonal information required to resolve the diagnosis within those contexts.

Finally, the selected features can be mapped to physiologically interpretable properties of the upper airway. The spectral centroid expands the distribution of energy across frequencies. Kurtosis and skewness capture resonance and asymmetry, respectively. Entropy represents signal complexity, and the zero-crossing rate indicates rapid oscillatory changes, reflecting the high-frequency spectral content associated with fine-scale flow disturbance and turbulent noise. The recurrence of these features across folds and sub-classifiers highlights their stability and robustness as markers of OSA during wakefulness. Overall, our findings imply that the combined contribution of inspiratory and expiratory features captures discriminative information. This simultaneous contribution underscores the importance of including both inspiration and expiration phases in the recording protocols.

### 4.2. Ensemble Fusion Strategies

Application of an ensemble learning approach was essential in boosting the model’s robustness as well as generalizability. Nine sub-classifiers were constructed using stratified subsets defined by anthropometric factors, namely sex, age, BMI, MpS, and smoking history. The resultant subgroup-specialized training allowed for each classifier to exploit the best relevant features in their sub-population, thus reducing feature relevance heterogeneity across the entire population. A probability-based voting scheme was adopted to combine all the viable sub-classifiers determined by the anthropometric features of individuals. This voting mechanism reduced abrupt transitions and provided smoother decision boundaries. In addition, it enabled the ensemble to exploit the varying confidence levels of sub-classifiers, reducing susceptibility to overfitting.

The ensemble fusion strategy resulted in an improved balanced accuracy (72.1 ± 2.0%) and a better sensitivity-specificity trade off compared with the STOP-Bang questionnaire, the previous AWakeOSA algorithm, and a global non-stratified baseline. The enhanced performance supports the hypothesis that anthropometric-based sub-classifiers enable the models to learn the acoustic and physiological variations associated with OSA within each sub-classifiers, which single global models fail to capture effectively. The leave-one-subgroup-out analysis also confirms the effectiveness of the fusion schema and the unique contribution of each sub-classifier to final classification.

These findings illustrate how the ensemble fusion leverages inter-subgroup diversity, which is an essential property for achieving robust and unbiased predictions. It not only mitigated class imbalance and subject heterogeneity, but also enhanced interpretability by revealing which anthropometric profiles contribute most to classification performance. The outcomes across multiple analysis confirm that the diversity and probabilistic integration of sub-classifiers have significant potential to improve generalization and good sensitivity-specificity, even with a noisy recording.

### 4.3. Algorithm Performance

The developed ensemble algorithm achieved an average balanced accuracy of 72.1%, with a high sensitivity of 84.3% and a relatively lower specificity of 59.9%. The AUC of 0.77 indicates a fair-to-good discriminative capacity. When compared to other awake OSA screening modalities, such as speech-based frameworks which report accuracies higher than 80% [31] or previous TBS algorithms using advanced machine learning in quiet environments [36,37], our performance appears lower. However, it is essential to consider the experimental setting; while mentioned studies were largely validated in soundproof and highly controlled environments, this study was conducted in a noisy clinical condition with an average SNR of approximately 10 dB. In such realistic conditions, the harmonic structures required for speech analysis are often masked, and high-complexity models are prone to overfitting ambient noise. Therefore, the 77.1% accuracy achieved here represents a robust performance baseline for real-world clinical deployment using inexpensive equipment. The results imply that the framework is reliable in detecting OSA during wakefulness in a noisy environment and exhibits stable classification performance for various fold partitions. Notably, the higher sensitivity compared to specificity implies that the system is more effective at detecting OSA cases than at accurately excluding non-OSA subjects. Our threshold optimization analysis (Figure 6) highlighted the critical trade-off inherent to this class imbalance. While increasing the decision threshold could balance sensitivity and specificity, this adjustment would result in a substantial reduction in sensitivity. In clinical settings, this trade-off is acceptable for a screening tool, as minimizing false negatives is important in the prevention of adverse effects from undetected OSA, even at the expense of the possible increase in false positives that require confirmatory PSG follow-up [53]. Therefore, maintaining the threshold at 0.5, despite the lower specificity, prioritizes the capture of at-risk individuals, ensuring that the framework functions effectively. The observed performance trends can be attributed to OSA-related physiology as well as the limitations of the methodology adopted. For instance, OSA individuals have significantly elevated values of upper airway collapsibility, as measured by both acoustic analysis of breathing sounds [54], and anthropometric techniques like acoustic pharyngometry [55], leading to increased sensitivity. In contrast, the subjects who do not meet the criteria for OSA but have intermediate risk factors such as increased BMI or larger neck circumference might manifest similar acoustic and anthropometric features, thus reducing specificity [56,57].

### 4.4. Error Analysis

The aggregated confusion matrix (Table 9) showed that the ensemble correctly classified 123 individuals with obstructive sleep apnea (OSA) and 28 without OSA and misclassified 26 instances of OSA (false negatives) and 19 instances of non-OSA (false positives). Examining the clinical and demographic characteristics of these groups provided important insight into the sources of the errors.

False negatives (*n* = 26) consisted of patients with OSA with lower AHI values (32.3 ± 15.5 events/hour) compared with the correctly classified OSA subjects (44.7 ± 28.0 events/hour). This group also had lower anthropometric risk factors, including reduced BMI (31.9 ± 5.2 kg/m^2^) and lower neck circumference (38.5 ± 2.8 cm). In addition, a majority of this population were females (76.9%), suggesting that sex-related differences in acoustic markers could have contributed to their misclassification. While these patients were diagnosed clinically with OSA, their acoustic and anthropometric features were indistinguishable from those of non-OSA subjects, thereby impairing the sensitivity of the classifier.

False positives (*n* = 19) had low AHI levels (6.3 ± 2.5 events/hour) that validated their classification as non-OSA; however, their BMI (32.3 ± 7.1 kg/m^2^) and neck size (40.3 ± 3.2 cm) were similar to those patients with OSA, with an average BMI (36.8 ± 7.1 kg/m^2^) and neck circumference (44.7 ± 4.9 cm). This anthropometric similarity probably led to the acoustic characteristics of these patients being incorrectly interpreted as those of OSA. A disproportionate number of these cases were male (63.1%), as expected for the known tendency for male airway anatomy to exhibit increased collapsibility in the absence of OSA. Overall, misclassifications were found to be consistently associated with participants having borderline phenotypes. These would include OSA patients with decreased anthropometric measurements and lower AHI values, as well as non-OSA participants with anthropometric features usually characteristic of OSA.

Non-OSA subjects with AHI values in the intermediate range (10 ≤ AHI ≤ 15), who were excluded from the training phase to ensure clearer separation between OSA and non-OSA classes, were evaluated separately as an independent test group. The majority of these subjects were classified as OSA subjects; the average was 39.16 ± 2.03% across four folds. This reduced performance was anticipated and reflects the inherent ambiguity of this AHI range which lies very close to the diagnostic threshold of OSA (AHI = 15). It is therefore debatable whether individuals with an AHI of 10–15 exhibit clinically meaningful pathological differences compared to those with an AHI of 15–20. Nevertheless, AHI remains the most widely used diagnostic metric for identifying patients requiring treatment. In contrast, we hypothesize that tracheal breathing sounds capture underlying physiological alterations associated with OSA more directly, rather than relying on a rigid threshold-based definition. It should further be noted that the BMI, age, and neck circumference of these participants were also at the borderline values between the corresponding groups; thus, the borderline nature of anthropometric parameters of these subjects could also be contributing to the low accuracy for this group’s classification.

In addition, recording instrumentation may have further impacted classification performance. A Primo EM273 capsule was used in this study, while a Sony ECM77B microphone was employed in our previous work [39]. The Sony ECM77B has been identified as an extremely effective omnidirectional electret condenser microphone characterized by its exceptionally low self-noise, high signal-to-noise ratio, wide and flat frequency response, and effective shielding through a balanced XLR output. Collectively, all of these features significantly contribute to its ability to record subtle physiological sounds while reducing the likelihood of interference from external sources. In contrast, the Primo EM273 capsule utilized in this application offers a miniature and affordable option, which demonstrates quite acceptable sensitivity accompanied by comparatively low levels of noise within its class. However, as a result of its unmounted three-wire configuration and quite minimal internal shielding, it is notably vulnerable to electrical disturbances resulting from electromagnetic forces, bias voltage variations, and acoustic resonance within the chamber. While the EM273 offers versatility, miniaturization, and compatibility with proprietary chamber configurations, the ECM77B offers better stability in producing cleaner output signal recordings. The higher levels of noise and harmonic artifacts observed in the present recordings, compared with the preceding sounds in the previous study [39], are likely attributable, at least in part, to these hardware differences.

### 4.5. Limitations and Future Directions

This study has several limitations. First, while the sample consisted of 196 individuals, the database contained a small number of non-OSA cases (*n* = 47), thus causing class imbalance and, unfortunately, affecting the training performance of the resultant subgroup classifiers due to the profound underrepresentation of the non-OSA individuals. The resulting lower specificity of the classifiers likely stemmed from the former finding. Second, the data quality was heterogeneous: seven participants were excluded due to low SNR and sub optimally sampled breathing phases, and 12 participants were rejected due to the presence of persistent harmonic noise. These exclusions reduced the statistical power, and the remaining database still contained recordings with persistent attendant noise and artifacts. Third, the poor representation of non-OSA subjects in certain sub-classifiers also reduces their performance in correctly defining class boundaries. For instance, high BMI subgroup includes only 11 non-OSA subjects, resulting in a training set of just 7 non-OSA samples per fold. While 1000 iterations were implemented to mitigate this by selecting only features that remain stable across resampled subsets, the limited pool of unique subjects still increases the risk of overfitting. Consequently, the model may memorize the specific acoustic traits of these individuals and be unable to capture the full acoustic variability inherent to non-OSA individuals. Fourth, a methodological limitation was the exclusion of subjects with intermediate OSA severity (10 ≤ AHI ≤ 15). While this exclusion was implemented to enhance separability and facilitate the learning of distinct acoustic features during the training phase, it limits the immediate generalizability of the finding to the full spectrum of clinical populations. Clinically, this “gray zone” represents a diagnostically challenging group. However, our framework was designed not merely to replicate AHI-based diagnostic thresholds, which can fluctuate due to night-to-night variability, but to detect underlying physiological phenotypes (AHI < 10 vs. AHI > 15), the sub-classifiers learn to identify the acoustic correlates of airway collapsibility.

Future work should focus on strengthening the generalizability and clinical applicability of the proposed framework, particularly for large-scale deployment in an uncontrolled home environment. Specifically, future work will focus on testing the model on the excluded intermediate group to evaluate whether their acoustic features align more closely with the non-OSA or OSA phenotypes, thereby offering a physiological assessment rather than a strictly count-based diagnosis. To ensure the robustness of the framework across diverse patient populations, future studies should expand the validation cohort to include wider range of anthropometric profiles and varying ambient noise conditions. In terms of algorithm refinement, employing sophisticated audio features such as wavelet scattering coefficients, modulation spectral descriptors, and complex quantities (such as entropy and detrended fluctuation analysis, used as complex numbers) can potentially reveal small characteristics of airway issues and non-linear airway dynamics that basic statistics may overlook. Furthermore, selecting characteristics by stability (such as employing Elastic Net stability selection or thinning by thinning informed by Shapley Additive Explanations (SHAP)-informed thinning) would ensure the predictors are stable and interpretable across varied groups. Lastly, the probability ensemble could be refined by adopting weighted fusion schemes, in which the sub-classifiers are weighted according to their cross-validated performance and adjusted to compensate for subgroup imbalances. In addition to these audio advances, the inclusion of additional physiological inputs such as oxygen saturation level, airflow resistance, chin motion, and speech recordings may give greater detail. There are significant cues in the audio regarding the shape and the resonance of the airway [48], as well as potential obstructions [34], and incorporating these features with lung and tracheal sounds may allow the model to better distinguish between different physiological conditions. Overall, these refinements aim to transition the current prototype into a scalable, noise-tolerant screening tool suitable for widespread population health monitoring.

## 5. Conclusions

Obstructive sleep apnea (OSA) is a common respiratory disorder that is largely undiagnosed, mainly due to the cost of and the limited accessibility of diagnostic equipment such as polysomnography. In this research, a classification system that combines respiratory sound recordings during wakefulness using a low-cost microphone was developed. Although this approach inherently accepts environmental noise, it provides an affordable and practical alternative for OSA screening. Using wavelet packet decomposition, acoustic features were extracted, and a Minimum Redundancy Maximum Relevance (MRMR) approach was used to reduce the number of features. To compensate for inter-individual variability, subgroup-specific classifiers stratified by sex, age, body mass index (BMI), and smoking history were trained, and their predictions were combined using a probability voting system, which further helped reduce the effect of class imbalance. Our suggested framework with the utilized microphone attained a balanced accuracy of 72.1%, sensitivity of 84.3%, and specificity of 59.9%. Since identifying individuals with OSA is more crucial than identifying false positives, a high percentage of sensitivity could compensate for the low specificity. These results indicate that despite substantial environmental noise, the combination of tracheal breathing recordings acquired during wakefulness when combined with the proposed ensemble framework, may be used as a viable and interpretable method for OSA screening. Future studies should include larger and more diverse populations to confirm generalizability. Advanced noise reduction and artifact removal techniques may lead to better signal quality and more reliable feature extraction. Adding complementary modalities, such as speech signals, could provide further insight into airway physiology and strengthen predictive performance. Finally, incorporating additional clinical or anthropometric variables, such as oxygen desaturation, time since smoking cessation, and airflow resistance, may also help ground the model in underlying physiology.

## Figures and Tables

**Table 1:** Demographic and clinical information of the study cohort. Data for age, BMI, and AHI are presented as mean ± standard deviation or n count (number of subjects). All other variables are reported as n count. Abbreviations and notations used are: n (sample size); age (in years); sex (male/female count); BMI (calculated as $kg/m^{2}$); MpS (Roman numerals I–IV denote the count for each class); and AHI (measured as number of events per hour).

Class	*n*	Age	Sex (M/F)	BMI	MpS (I, II, III, IV)	Smoking History (Never, Former, Current)	AHI
OSA	149	48.1 ± 12.0	(99, 50)	35.9 ± 8.9	(32, 26, 38, 53)	(85, 37, 28)	42.6 ± 26.7
Non-OSA	47	44.1 ± 13.0	(19, 28)	31.9 ± 6.8	(10, 11, 17, 9)	(34, 6, 7)	5.7 ± 2.6

**Table 2:** Name of the features extracted from each frequency sub-band.

Category	Feature List
Statistical	1. Mean, 2. Variance, 3. Std Deviation, 4. Energy, 5. Median
Higher-Order	6. Skewness, 7. Kurtosis
Temporal	8. Zero-Crossing Rate
Spectral	9. Centroid, 10. Spread, 11. Skewness, 12. Kurtosis, 13. Entropy
Complexity	14. Shannon Entropy, 15. Tsallis entropy, 16. Rényi Entropy

**Table 3:** Distribution of Study Subjects Stratified by Clinical and Demographic Subgroups.

Subgroup	OSA	Non-OSA	Ratio *
Male	99	19	5.21
Female	50	28	1.79
High Age	64	18	3.56
Low Age	85	29	2.93
High BMI	66	11	6.00
Low BMI	83	36	2.31
High MpS	91	26	3.50
Low MpS	58	21	2.76
Non-Current Smokers	121	40	3.03

* Ratio is defined as the number of OSA subjects over non-OSA subjects.

**Table 4:** Summary of the consistently selected acoustic features across outer cross-validation folds that appeared in at least three out of four folds for each anthropometric sub-classifier. Abbreviations: MI: Mouth Inspiration; ME: Mouth Expiration; NI: Nose Inspiration; NE: Nose Expiration.

Feature No.	Sub-Classifier	Descriptor	Maneuver	Frequency Range (Hz)
9	Male	Spectral Centroid	MI	0–172
473	Male	Spectral Centroid	NI	1550–1722
276	Male	Kurtosis	ME	1206–1378
412	Female	Spectral Entropy	NI	861–1033
410	Female—Low BMI	Spectral Kurtosis	NI	861–1033
616	High Age	Median	NE	1378–1550
373	High Age	Standard Deviation	NI	517–689
522	Low Age	Spectral Kurtosis	NE	344–517
362	High BMI	Spectral Kurtosis	NI	344–517
113	Low BMI	Mean	MI	1206–1378
628	High MpS	Kurtosis	NE	1550–1723
599	Low MpS– Non-Current Smokers	Zero-Crossing Rate	NE	1206–1723

**Table 5:** Classification performance of the proposed OSA detection algorithm across 4-fold cross-validation using probability-based voting. Reported metrics include balanced accuracy (average of sensitivity and specificity), accuracy, sensitivity, specificity, and AUC. Values are given for each fold and as mean ± standard deviation across folds.

Metric	Fold 1	Fold 2	Fold 3	Fold 4	Average
Balanced Accuracy	74.5	68.4	72.4	69.6	72.1 ± 2.0
Accuracy	75.5	73.5	79.6	79.6	77.1 ± 3.1
Sensitivity	76.3	78.4	86.5	89.2	84.3 ± 5.8
Specificity	72.7	58.3	58.3	50.0	59.9 ± 9.4
AUC	0.80	0.78	0.74	0.74	0.77 ± 0.01

**Table 6:** Classification performance of the proposed OSA detection algorithm across 4-fold cross-validation using majority voting. Reported metrics include balanced accuracy (average of sensitivity and specificity), accuracy, sensitivity, and specificity. Values are given for each fold and as mean ± standard deviation in percentage across folds.

Metric	Fold 1	Fold 2	Fold 3	Fold 4	Average
Balanced Accuracy	78.5	66.9	72.4	66.9	71.2 ± 5.5
Accuracy	81.6	75.5	79.6	75.5	78.1 ± 3.1
Sensitivity	84.2	83.8	86.5	83.8	84.6 ± 1.3
Specificity	72.7	50.0	58.3	50.0	57.8 ± 10.7

**Table 7:** Performance of the ensemble framework under leave-one-subgroup-out (LOSO) fusion. Each row shows results when predictions from the corresponding sub-classifier were excluded from the ensemble decision. Reported metrics included balanced accuracy, accuracy, sensitivity, and specificity (%).

Sub-Classifier	Balanced Accuracy	Accuracy	Sensitivity	Specificity
Male	68.9	74.0	79.2	57.8
Female	67.4	73.5	79.2	55.7
High Age	70.5	77.0	83.3	57.8
Low Age	71.2	77.0	82.6	59.9
High BMI	68.2	73.5	78.5	57.8
Low BMI	69.5	76.5	83.3	55.7
High MpS	71.2	77.0	83.6	59.9
Low MpS	70.2	76.5	82.6	57.8
Non-Current Smokers	68.3	77.6	85.2	51.3

**Table 8:** Comparison of different diagnostic methods with our proposed ensemble framework. Reported metrics include balanced accuracy, accuracy, sensitivity, and specificity, expressed as mean ± standard deviation in percentage across four folds.

Algorithm	Balanced Accuracy	Accuracy	Sensitivity	Specificity
STOP-Bang [28]	60.9 ± 3.4	76.9 ± 2.2	93.6 ± 2.5	28.3 ± 3.1
AWakeOSA [40]	48.4 ± 1.9	44.9 ± 6.1	37.8 ± 3.6	59.1 ± 5.7
Global Bagged Trees	56.6 ± 4.7	78.6 ± 1.2	98.7 ± 1.6	14.6 ± 10.5
**Proposed Framework**	**72.1 ± 2.0**	**77.1 ± 3.1**	**84.3 ± 5.8**	**59.9 ± 9.4**

**Table 9:** Clinical and demographic characteristics of subjects stratified by classification outcome (correct OSA, correct non-OSA, false negative, false positive). Continuous variables are expressed as mean ± standard deviation; categorical variables are shown as percentages or count.

Parameters	Correct OSA	Correct Non-OSA	False Negative	False Positive
*n*	123	28	26	19
Male (%)	75.6	25	23.1	63.1
Age	47.7 ± 12.0	43.9 ± 13.3	50.2 ± 2.8	46.3 ± 12.9
BMI (kg/m^2^)	36.8 ± 7.1	31.7 ± 6.8	31.9 ± 5.2	32.3 ± 7.1
Current Smokers (%)	20.3	21.4	22.5	5.3
Neck Circumference	44.7 ± 4.9	38.3 ± 3.8	38.5 ± 2.8	40.3 ± 3.2
MpS	(25, 22, 34, 42)	(5, 8, 10, 5)	(7, 4, 4, 11)	(5, 3, 7, 4)
Snoring (%)	96.8	85.7	92.3	84.2
Receding Mandible	(4, 116, 3)	(3, 23, 2)	(0, 23, 3)	(1, 71, 1)
AHI	44.7 ± 28.0	5.3 ± 2.6	32.3 ± 15.5	6.3 ± 2.5

## Data Availability

Data is available for sharing upon request to the corresponding author, Zahra Moussavi.

## Abbreviations

The following abbreviations are used in this manuscript:

OSA	Obstructive sleep apnea
BMI	Body mass index
MpS	Mallampati score
PSG	polysomnography
AHI	Apnea-Hypopnea index
HSAT	Home sleep apnea testing
ESS	Epworth Sleepiness Scale
TBS	Tracheal breathing sound
SNR	Signal-to-noise ratio
WPD	Wavelet packet decomposition
mRMR	Minimum redundancy maximum relevance
AUC	Area under the curve
LOSO	Leave-one-subgroup-out
EFL	Expiratory flow limitation
